# Administration of Nirsevimab for RSV Prophylaxis in Infants: A Comprehensive Review

**DOI:** 10.3390/vaccines13050470

**Published:** 2025-04-27

**Authors:** Pan-Pan Wu, Fang-Rui Ding

**Affiliations:** 1 Department of Neonatology, Tianjin Central Hospital of Obstetrics and Gynecology, Tianjin 300100, China; wupanpan356@126.com; 2 Tianjin Key Laboratory of Human Development and Reproductive Regulation, Tianjin 300100, China; 3 Department of Neonatology, Nankai University Maternity Hospital, Tianjin 300100, China

**Keywords:** respiratory syncytial virus, infants, nirsevimab, monoclonal antibodies

## Abstract

Respiratory syncytial virus (RSV) is the primary etiological agent responsible for lower respiratory tract infections (LRTIs) and hospitalizations among infants. Nirsevimab, a novel monoclonal antibody (mAb), offers sustained protection against RSV for a minimum of 5 months in neonates and young children. Extensive clinical trials and real-world evidence have demonstrated that nirsevimab significantly mitigates the incidence and severity of RSV infections in infants, while exhibiting favorable safety profiles and cost-effectiveness. Regulatory authorities in multiple countries have approved nirsevimab, and its implementation is progressively expanding across various healthcare settings. However, several critical issues require further attention. Specifically, a more in-depth investigation into the long-term efficacy and benefits of nirsevimab across diverse populations, particularly neonates, is essential. Additionally, accelerating the introduction and administration of nirsevimab in developing countries remains imperative. Thus, this review comprehensively summarizes the administration of nirsevimab in infants to facilitate its broader application.

## 1. Background

### 1.1. Introduction of RSV

Respiratory syncytial virus (RSV) is a single-stranded, enveloped RNA virus with a negative-sense genome, classified within the Pneumoviridae family. First isolated in 1955, RSV underwent detailed characterization beginning in 1981. As the type species of the Pneumovirus genus (Pneumovirinae subfamily, Paramyxoviridae family, order Mononegavirales), human RSV is divided into two antigenically distinct subgroups, A and B, which exhibit genome-wide sequence divergence. The Pneumovirinae subfamily also includes the Metapneumovirus genus, comprising human and avian metapneumoviruses, while the other Paramyxovirinae subfamily contains a wider array of pathogens, such as parainfluenza viruses, mumps virus, measles virus, Nipah virus, Hendra virus, and other emerging members [1]. Structurally, RSV has 10 genes and encodes 11 proteins, making it more complex than most Paramyxovirinae members. These proteins include Surface glycoproteins: Fusion (F) and attachment (G) proteins, which serve as the primary viral neutralization and protective antigens; M1, M2-1, and M2-2 matrix proteins, NS1 and NS2 virion proteins, SH protein, and N, D, L nucleotide capsule proteins. The G glycoprotein facilitates viral attachment to host cells, while the F glycoprotein mediates membrane fusion, enabling viral entry and subsequent syncytia formation [1,2]. This pathogen demonstrates a global seasonal distribution, significantly shaped by geographic variations [3]. In most Southern Hemisphere nations, RSV activity peaks between March and June, whereas in Northern Hemisphere regions, it is most prevalent from September to December [4]. RSV stands out as the primary cause of acute lower respiratory tract infections (LRTIs) in infants younger than 1 year and remains a leading reason for hospitalizations among children up to 2 years of age [5,6].

### 1.2. Health Burden Caused by RSV

A comprehensive evaluation of the global disease burden revealed that in 2019, RSV was responsible for 33 million LRTIs, representing 22% of all LRTI cases. It also led to 3.6 million hospitalizations and 101,400 fatalities among children under 5 years old, and accounting for 2% of deaths in this population. Notably, for infants between 0 and 6 months, RSV caused 6.6 million LRTIs, 1.4 million hospitalizations, and 13,300 deaths. Furthermore, RSV accounted for 3.6% of mortality in infants aged 28 days to 6 months [5]. Research based on population studies indicates that the highest rate of RSV-related hospitalizations occurs in infants aged 0–3 months, with an incidence ranging from 24.7 to 31.7 per 1000 child-years [7]. In a RESCEU study spanning five European countries, which included 9154 healthy full-term infants (gestational age [GA] ≥37 weeks), it was found that 26.2% of these infants experienced RSV infections during their first year of life, as confirmed by multiple diagnostic methods. The proportion of RSV infections necessitating medical attention was 14.1%, while the hospitalization rate due to RSV stood at 1.8%. Notably, among the 145 infants hospitalized for RSV-related complications, 8 required admission to pediatric intensive care units (PICU), representing 5.5% of these cases. Furthermore, 60% of the hospitalizations occurred in infants under 3 months old [8].

RSV-LRTIs during early childhood have been linked to subsequent long-term respiratory complications, including recurrent LRTIs, wheezing, asthma, and reduced lung function [9]. A 2013 meta-analysis involving 82,008 participants found that children who had experienced RSV-LRTI were 3.84 times more likely to develop wheezing and asthma compared to those without such infections [10]. In contrast, a 2020 meta-analysis of 11,195 infants showed that the use of monoclonal antibodies (mAbs) for preventing RSV bronchiolitis did not significantly reduce the risk of subsequent recurrent wheezing or asthma (relative risk [RR] 0.60) [11]. A systematic review of 41 studies showed that RSV-infected infants had an odds ratio of 3.05 for recurrent wheezing up to 36 months and 2.95 for asthma up to 12 years [12]. Furthermore, research has shown that diminished lung function in early life is associated with a higher risk of cardiovascular events and premature mortality in adulthood [13]. A prospective, nationally representative cohort study in Great Britain, spanning the entire lifespan, demonstrated that LRTIs during early childhood (under 2 years of age) were independently associated with nearly double the risk of premature mortality from respiratory diseases (hazard ratio 1.93; 95% confidence interval [CI]: [1.10–3.37]), even after adjusting for adult smoking and other potential confounders [14].

### 1.3. Treatment of RSV-Related LRTIs

Considering the significant public health implications and limited therapeutic options, preventing RSV disease has become an urgent global health priority [15]. Treatment for RSV-LRTI is primarily supportive, with preventive measures such as isolation and good hygiene forming the cornerstone of management. Therefore, preventing severe RSV infections in high-risk patients is paramount. According to the WHO, strategies for preventing RSV infection in children include both passive administration of immunoglobulins and active immunization. In the 1960s, infants were intramuscularly injected with a formalin-inactivated RSV vaccine (FI-RSV) that was formulated with alum. However, the vaccine proved ineffective. Studies reported in 1969 showed that in age-matched children with RSV infection, the incidence of severe RSV cases was significantly higher among those who received FI-RSV (44%) compared to those who received a placebo (6%). This discrepancy led to the deaths of two infants who were administered FI-RSV [16,17,18]. Over subsequent decades, RSV vaccine development faced multiple challenges, including safety concerns, our incomplete understanding of the immune response to RSV, and its relationship to the severity of clinical disease [19,20]. Currently, passive immunization is the only available option for infants, which can be achieved either through direct antibody administration or maternal immunization during pregnancy. To date, several vaccine candidates for children are under development, but none have been approved for use yet. Abrysvo is the only licensed vaccine designed to prevent LRTI caused by RSV in pregnant women between 24 and 36 weeks of gestation, thereby reducing the risk of LRTI and severe RSV-related illness in neonates and infants up to 6 months after birth [21,22]. However, a US research report indicates that the vaccination rate for Abrysvo among pregnant individuals was only 32.6% [23].

### 1.4. Prophylaxis Against RSV Infection in Infants

Prior to 2023, palivizumab, a humanized mAb targeting RSV, was the only available prophylactic option. A 2014 systematic review, which encompassed seven RCTs, four open-label non-comparative studies, and eight prospective observational studies, analyzed over 42,000 high-risk infants and children from 34 countries. The findings indicated that palivizumab reduced RSV-related hospitalizations by 39% to 78%, with an overall odds ratio of 0.41 favoring palivizumab prophylaxis over placebo [24]. In a more recent 2023 study based on data from a large, multicenter, prospective, observational cohort in the Netherlands, involving preterm infants within their first year of life, palivizumab prophylaxis was shown to significantly lower infection rates among very preterm infants (≤32 weeks gestational age, wGA). Specifically, the infection rate was 18.9% in the prophylaxis group compared to 48.3% in the non-prophylaxis group. For infants with a GA between >32 and ≤36 weeks, the infection rate in the non-prophylaxis group was 55.4%, aligning closely with the rate observed in the non-prophylaxis group of infants with a GA of ≤32 weeks [25]. Furthermore, a cost-effectiveness analysis for palivizumab in premature infants without chronic lung disease revealed higher expected costs for palivizumab prophylaxis compared to no prophylaxis [26]. Consequently, due to its extremely high cost and the necessity for multiple monthly doses during the RSV season, palivizumab’s use has been primarily limited to high-risk infants, representing less than 5% of all infants in well-resourced healthcare settings [27].

Nirsevimab (marketed as Beyfortus) is a recombinant human IgG1κmonoclonal antibody jointly developed by AstraZeneca and Sanofi Pasteur for the prevention of RSV infection. This long-acting monoclonal antibody targets a highly conserved epitope spanning the F1 and F2 subunits of the RSV fusion (F) protein, locking it in the prefusion conformation to effectively block viral entry into host cells. First approved in the European Union on 3 November 2022, nirsevimab has since obtained regulatory approval in various regions, including the United States and Australia, and has been introduced in these areas. Unlike palivizumab, a single dose of nirsevimab offers infants continuous protection against RSV for at least 5 months, covering an entire typical RSV season [28,29,30,31,32,33,34,35].

## 2. Methods

**Figure 1:**
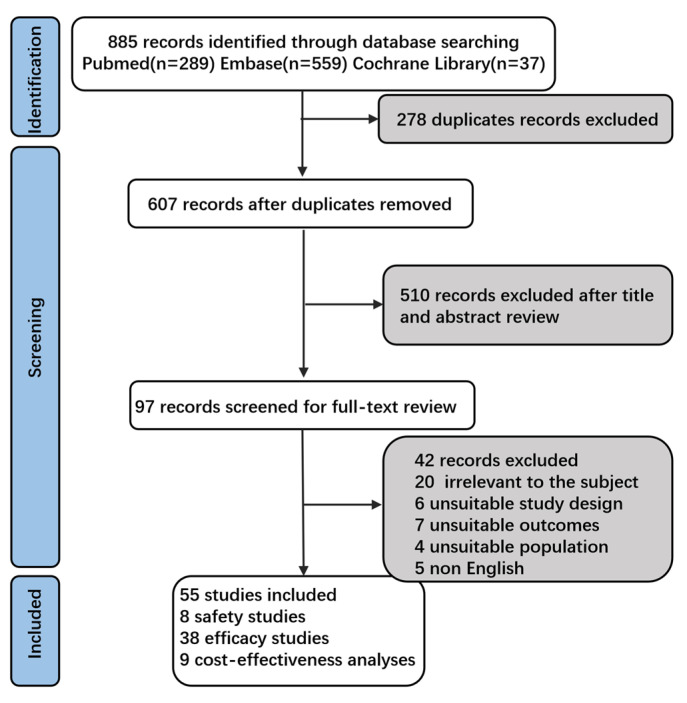
Flowchart of study identification and inclusion. Initial database searches using ‘nirsevimab’ as the primary keyword yielded 885 records. After removing 278 duplicates, 607 records underwent title and abstract screening, excluding 510 irrelevant studies. Full-text review was performed on 97 articles, with 55 studies meeting the final inclusion criteria.

This comprehensive systematic review aims to present the safety, effectiveness, cost-effectiveness, application recommendations, and implementation challenges of nirsevimab in infants. To identify eligible studies, a thorough search of the PubMed, EMbase and Cochrane Library databases was conducted from its inception to 20 February 2025. We updated our search on 17 April 2025. All references obtained using ‘nirsevimab’ as a keyword were included, resulting in an initial reference list for further selection. Based on this search strategy, 289 reports were retrieved from PubMed, 559 articles from EMbase, and 37 articles from the Cochrane Library. Studies focusing on the safety, effectiveness, cost-effectiveness, and immunization challenges of nirsevimab for infants were selected. The following studies were excluded: (1) duplicate publications; (2) reviews, editorials, conference papers, case reports, or animal studies; and (3) those not relevant to safety, efficacy, or cost-effectiveness (Figure 1).

## 3. Summary of Results and Discussion

Based on the search strategy, this review includes evidence from 8 safety studies, 38 efficacy studies, and 9 cost-effectiveness analyses across eight countries: the US, Spain, Saudi Arabia (KSA), the UK, Italy, Japan, Canada, and The Netherlands. Safety data from real-world studies demonstrate no serious adverse events (SAEs) to date. Efficacy findings, supported by pivotal trials and real-world evidence (including meta-analyses, population-based studies, and case-control designs), consistently show protection against RSV-LRTI. Cost-effectiveness analyses primarily employed static models of birth cohorts, with limited dynamic modeling. According to GRADE guidelines [36], there was high confidence in nirsevimab’s safety, efficacy, and cost-effectiveness for preventing RSV-LRTI.

## 4. Safety and Adverse-Event Profile of Nirsevimab

In clinical trials, the incidence of adverse events (AEs) was comparable between the nirsevimab and placebo group, with no SAEs attributed to the nirsevimab group. The most frequently reported side effects within the first 7–14 days post-administration were rash, fever, and injection site reactions (including erythema, swelling, and pain). Previously published studies have underscored rare yet significant adverse effects, including hypersensitivity reactions and thrombocytopenia [37]. In both the Phase 2B (NCT02878330) and Phase 3 (NCT03979313) trials, the nature and incidence rates of AEs were similar between the nirsevimab and placebo groups. Most AEs were classified as mild to moderate in severity (Grade 1 or 2). In the Phase 2B trial, the rate of SAEs in the nirsevimab group was 11.2%, with a 0.5% incidence for events of special interest. For the placebo group, these figures stood at 16.9% and 0.6%, respectively. Overall, there were five fatalities: two occurring in the nirsevimab group and three in the placebo group. In the Phase 3 trial, the SAE incidence rates were 6.8% for the nirsevimab group and 7.3% for the placebo group. One participant in the nirsevimab group experienced a Grade 3 generalized macular rash 6 days after injection, which resolved on its own within 20 days without any specific treatment. Three deaths occurred among nirsevimab recipients, all on or after day 140. In both clinical trials, all SAEs, including fatalities, were determined to be unrelated to the investigational drug. Notably, no instances of anaphylaxis or other severe allergic reactions were reported [38,39] (Table 1). In the Phase 2/3 MEDLEY trial (NCT03959488), 925 high-risk or premature infants (≤35 wGA) proceeded into a second RSV season. These participants were categorized into two main groups: those born prematurely and those at high risk. Within each category, infants were further assigned to either the palivizumab or nirsevimab treatment groups. The incidence of AEs was comparable between the groups. There were five fatalities in the nirsevimab group—two among preterm infants and three among high-risk infants. One death occurred in the high-risk palivizumab group. Investigators considered that none of these deaths were related to the treatments administered. Two specific AEs were observed in the nirsevimab group: one case of heparin-induced thrombocytopenia in an infant with congenital heart disease and one instance of a maculopapular rash in a preterm infant who had received a placebo dose [40]. In the second RSV season, no rise was observed in the number of medically attended RSV-LRTIs or in disease severity among the infants from the Phase 3 Melody trial. Additionally, there was also no indication of antibody-dependent enhancement of infection [41].

Given the novelty of nirsevimab, real-world evidence regarding its safety remains limited, with findings still emerging. Currently, the only relevant studies have been reported in the HARMONIE trial and investigations from Italy and France. The HARMONIE trial was a multicenter study conducted across Germany, France, and the UK. It involved 8058 infants who were born with GA exceeding 29 weeks and were entering their first RSV season. According to the trial results, treatment-related AEs were observed in 2.1% of infants in the nirsevimab group. Four infants experienced specific AEs of interest: one case each of drug reactions, allergic dermatitis, and maculopapular rash. All these events were categorized as either grade 1 or grade 2 in terms of severity. Additionally, one infant developed a grade 3 SAE—infantile spasms, also referred to as West syndrome—23 days after receiving nirsevimab. This event could not be definitively ruled out as potentially related to the trial treatment. In line with the clinical trial findings, medically attended AEs occurred at similar rates in both the nirsevimab and control groups, and no fatalities were reported [42]. In an Italian cohort study conducted in the Valle d’Aosta Region, a total of 484 individuals participated, among whom 292 (65%) received nirsevimab prophylaxis. AEs that were noted within 2 weeks after administration usually appeared within 48 h following treatment and these effects were predominantly mild and short-lived, typically resolving within 1 to 2 days. Reported AEs included fever in 6.5% of participants, local reactions at the injection site in 4%, and persistent crying in 0.4%. None required additional medical attention, and no SAEs were reported [43]. In a French longitudinal, prospective, single-center cohort study of 1730 infants, at least one SAE occurred in 9.4% of the nirsevimab group and 10.3% of controls, with no treatment-related events reported in either group [44].

## 5. Effectiveness of Nirsevimab for RSV Infection

Several large-scale clinical studies have confirmed the effectiveness and safety of nirsevimab in preventing RSV-LRTIs. In Phase 1b/2a trials, it was observed that peak serum concentrations of nirsevimab were attained within 1 week after administration, with over 90% of infants sustaining high mean serum levels for up to 5 months following treatment [45]. Three critical clinical trials evaluated the efficacy of nirsevimab in preventing RSV infections. In a Phase 2b trial, a total of 1453 preterm infants with GA ranging from 29 to 34 weeks were enrolled across 164 sites in 23 countries, spanning both hemispheres. In this research, 969 infants were administered a single intramuscular dose of 50 mg nirsevimab before the RSV season began, whereas 484 infants received a placebo. Compared to the control group, the nirsevimab group experienced a 70.1% decrease in the incidence of medically attended RSV-LRTIs. Furthermore, the risk of RSV-LRTI hospitalizations was reduced by 78.4% [38]. The Phase 3 clinical trial encompassed 1490 infants with a GA of at least 35 weeks. This research was conducted across 150 sites in 20 Northern Hemisphere countries and at 10 locations in 1 Southern Hemisphere country. At the onset of the RSV season, 994 infants received nirsevimab, while 496 were given a placebo. Unlike the Phase 2B trial, infants with weight less than 5 kg were administered a single 50 mg dose of nirsevimab, whereas those weighing 5 kg or more received a single 100 mg dose. Compared to the placebo group, the nirsevimab group experienced a 74.5% reduction in medically attended RSV-LRTIs and a 62.1% decrease in hospitalizations due to RSV-LRTIs [39]. When integrating data from both the Phase 2B and Phase 3 trials, nirsevimab demonstrated an overall effectiveness of 79.5% in preventing medically attended RSV-LRTIs, 77.3% in preventing RSV-LRTIs requiring hospitalization, and 86.0% in preventing very severe RSV-LRTIs—defined as cases needing supplemental oxygen or intravenous fluids after hospital admission for medically attended RSV-LRTI. No deaths related to RSV were reported in either trial. Additionally, there was no significant difference in the incidence of SAEs between the nirsevimab and placebo groups [46]. The HARMONIE study took place across France, the UK, and Germany between 8 August 2022 and 28 February 2023. This research involved infants who were one year old or younger, with a minimum GA of 29 weeks. A total of 8058 infants were randomly allocated to either receive nirsevimab (4037 infants) or undergo standard care (4021 infants). The findings indicate that nirsevimab was 75.7% effective in preventing extremely severe RSV-LRTI, specifically those cases necessitating supplemental oxygen because of an oxygen saturation level under 90%. Furthermore, nirsevimab significantly reduced hospitalizations caused by RSV-LRTI: by 89.6% in France, 83.4% in the UK, and 74.2% in Germany. These outcomes were especially notable among infants aged 0–3 months [42] (Table 2).

Clinical trials demonstrated substantial reductions in RSV-LRTI incidence and hospitalizations following a single intramuscular administration of nirsevimab, accompanied by a favorable safety profile. Additionally, its real-world effectiveness was investigated. Early real-world evidence from the 2023–2024 RSV season in the United States and Europe indicated that nirsevimab reduced hospitalizations due to RSV-LRTIs by 70% to 90% [28,29,32,33,37]. Multiple meta-analyses consistently demonstrate nirsevimab’s effectiveness in reducing RSV-related complications in infants. A comprehensive analysis of 13 studies (*n* = 45,238) combining five RCTs, seven observational studies, and one health authority set of data showed 88.4% efficacy against RSV hospitalizations [47]. Supporting evidence includes (1) a network meta-analysis (15 RCTs, *n* = 18,395) showing significant reductions per 1000 infants—123 fewer RSV-LRTIs, 54 fewer hospitalizations, and 59 fewer oxygen requirements [48]; (2) a multinational analysis (28 studies) demonstrating strong protection against severe outcomes (hospitalization OR 0.14, ICU admission OR 0.17) [49]; and (3) preterm infant data (*n* = 7347) showing 75% lower RSV-LRTI risk (OR 0.25) [50]. Additionally, a random-effects meta-analysis of two trials (data through June 2022) confirmed that a preseason nirsevimab prophylaxis significantly reduced medically attended RSV infections (RR 0.26) and hospitalizations (RR 0.24) versus a placebo, with comparable safety profiles for fatal adverse events (RR 0.78) and adverse events of special interest (RR 0.92) [51].

The clinical benefits of nirsevimab have been further corroborated by real-world implementation data from multiple countries. Spain introduced universal prophylaxis against RSV into its national immunization program for all infants born on or after 1 April 2023. An initial analysis of data from nine hospitals in Valencia, Murcia, and Valladolid revealed that the population-based coverage of nirsevimab varied between 78.7% and 98.6%, with variations depending on the hospital. The efficacy of nirsevimab in preventing RSV-LRTI hospitalizations was observed to range between 69.3% and 97.0% [28]. A population-based longitudinal study was conducted following an immunization campaign with nirsevimab in Galicia, Spain, which took place from 25 September 2023, to 31 March 2024. This campaign initially focused on three groups of infants: those born during the campaign period (seasonal group), infants under 6 months old (catch-up group), and those at high risk. A total of 9408 eligible infants received nirsevimab, including 6220 in the catch-up group and 3188 in the seasonal group. Among the high-risk infants, 348 out of 360 were administered nirsevimab. Based on data from both the seasonal and catch-up groups, nirsevimab showed an efficacy of 86.9% in preventing severe RSV-LRTIs requiring oxygen support, 82.0% for RSV-LRTI hospitalizations, 69.2% for all-cause LRTI hospitalizations, and 66.2% for all-cause hospitalizations. To prevent one RSV-LRTI hospitalization, 25 individuals needed to be immunized [29]. In a cohort study conducted in Spain, nirsevimab was administered to 1177 newborns within 7 days of birth, demonstrating an estimated effectiveness of 88.7% in preventing RSV hospitalizations; this intervention prevented one hospitalization for every 15.3 infants treated [30]. A study in Luxembourg analyzed data from two consecutive RSV seasons and found that hospitalizations among children under the age of 5 dropped by 38% from 2023 to 2024, decreasing from 389 cases to 241 cases. Moreover, hospitalizations for infants younger than 6 months plummeted by 69%, from 232 cases to 72 cases [33] (Table 3). In a French case-control test-negative design study, 288 infants aged less than 1 month or less than 5 months who were at high risk were enrolled from 20 PICUs between September 2023 and January 2024. Nirsevimab demonstrated an effectiveness of 75.9% against severe RSV-LRTIs requiring PICU admission. In sensitivity analyses, its effectiveness was 80.6% and 80.4%, respectively [34]. In a US retrospective cross-sectional study evaluating PICU utilization with or without RSV from January 2017 to June 2023, it was found that 11.4% of 119,782 PICU visits were associated with RSV, and 38.6% of these RSV-associated cases were eligible for RSV prevention. The analysis projected that if 65% to 85% of eligible children were immunized against RSV, it would result in a decrease in PICU visits by 2.1% to 2.8% and a reduction in PICU days by 4.5% to 5.9% [52].

Currently, data regarding the efficacy of nirsevimab in infants within outpatient settings remain limited. A test-negative case-control study utilizing data from the Yale-New Haven Health System included 3090 infants with a median age of 6.7 months. The study demonstrated that nirsevimab had an efficacy of 68.4% against RSV infections requiring medical intervention. Specifically, its efficacy was 61.6% for outpatient visits, 80.5% for RSV-related hospitalizations, and 84.6% for severe RSV outcomes necessitating intensive care or high-flow oxygen therapy. Protective efficacy against all-cause LRTIs and all-cause LRTI-related hospitalizations was observed exclusively during the peak 2-month period of the RSV season. The effectiveness rates were 49.4% for all-cause LRTIs and 79.1% for LRTI-related hospitalizations [67]. Another test-negative case-control study using data from the PARI network, which included 883 outpatients under 12 months of age, demonstrated an adjusted effectiveness of nirsevimab against RSV-bronchiolitis of 79.7% [68]. Additionally, a Spanish study among infants younger than 10 months showed an overall effectiveness of nirsevimab of 75.8%, and 80.2% in the catch-up group in primary care (PC) settings [69] (Table 3). These studies highlight the significance of outpatient catch-up immunization using nirsevimab in alleviating the strain on primary healthcare services.

Nirsevimab continues to be an effective strategy for mitigating the impact of RSV on healthcare resource use over a 5-month period. A prospective, dynamic, population-based cohort study was conducted in the Madrid region of Spain, involving 33,859 infants born between April and December 2023 who received nirsevimab immunization. These infants contributed to a total study population of 37,617. The infants were monitored throughout the 2023–2024 RSV season. During this period, there were 4100 PC visits, 1954 emergency room (ER) visits, and 509 hospital admissions, with 82 requiring ICU admission. Nirsevimab demonstrated an effectiveness of 93.6% at 30 days and 87.6% at 150 days in preventing RSV hospitalizations. Additionally, it was 94.4% effective at 30 days and 92.1% effective at 90 days in preventing ICU admissions [70]. In an initial evaluation of universal nirsevimab prophylaxis for high-risk infants and those under 6 months old in the Valencian Community, Spain, during the 2023–2024 RSV season, the coverage rate of nirsevimab administration was 88.5% among more than 27,000 infants. Nirsevimab demonstrated an effectiveness of 73.7% in preventing RSV infections. Hospitalization rates for acute respiratory infections were approximately halved in vaccinated infants compared to unvaccinated ones (0.9% vs. 1.6%, respectively) [71]. Additional research is crucial to assess the efficacy of nirsevimab in various settings, at different dosages, for mild RSV infections, and across diverse populations, especially among individuals with pre-existing medical conditions.

## 6. Cost-Effectiveness of Nirsevimab for RSV Prophylaxis

In addition to the clinical health burden, infections of RSV also entail substantial economic costs. Although nirsevimab demonstrates high efficacy in immunoprophylaxis, it is crucial to identify the most cost-effective strategy that maximizes value for money. According to the Advisory Committee on Immunization Practices (ACIP) subcommittee Work Group, for an infant in their first RSV season, the base case cost per quality-adjusted life year (QALY) saved is USD 102,811 when nirsevimab is administered at a price of USD 445 per dose. This price is derived from the average of the list price ( USD 495) and the Vaccines for Children (VFC) program price (USD 395) [72].

To assess the effects of nirsevimab on both outcomes and costs for infants in their first RSV season, static model analyses were performed in countries including the United States, Spain, and Saudi Arabia (Table 4). These analyses compared a universal infant immunization approach using nirsevimab against the standard of practice (SoP). Infants in the nirsevimab cohort received a single dose either at birth or at the onset of the RSV season. Conversely, the SoP cohort involved monthly administrations of palivizumab during the RSV season (up to five doses) for eligible infants, while ineligible preterm and full-term infants did not receive any prophylaxis. The infants were classified into three categories: full-term and/or late preterm infants, preterm infants, and palivizumab-eligible infants. The U.S. study estimated that universal nirsevimab immunization could decrease acute RSV-LRTIs by 290,174 cases, reduce hospitalizations by 24,986, and cut healthcare expenditures by USD 612 million (2021 USD) [73]. In Spain, it was anticipated that nirsevimab would prevent 97,157 PC visits (a 64.0% reduction), 24,789 ER visits (63.9%), 8185 hospitalizations (63.5%), 869 PICU admissions (61.5%), and 9 inpatient deaths (52.6%). This would result in healthcare cost savings of EUR 47.8 million (62.4%) [74]. In Saudi Arabia, universal nirsevimab prophylaxis was projected to prevent hospitalizations by 58%, which includes a 58% reduction in PICU admissions and mechanical ventilation episodes. Additionally, it reduced ER visits and PC visits by 53% each, as well as decreasing recurrent wheezing episodes by 58%, and preventing eight deaths, resulting in total healthcare cost savings of SAR 274–343 million [75]. Overall, compared to the SoP, nirsevimab immunization is expected to enhance infant health outcomes and decrease national healthcare expenditures. In current modeling studies, there is a lack of standardization. In 2023, a study evaluated the efficacy of nirsevimab by employing two distinct models for infants aged 0–11 months during their initial RSV season in the United Kingdom. The static model estimated that nirsevimab could prevent 16,255 (66%) hospitalizations and 102,633 (64%) general practitioner (GP) visits. In contrast, the dynamic transmission model estimated reductions of 5852 (28%) hospitalizations and 5108 (29%) GP visits [76]. Additionally, cost-effectiveness analyses from the UK, Italy, the Netherlands, Canada, and Japan further confirm that nirsevimab alleviates both the health and economic burden of RSV in infants [77,78,79,80,81,82] (Table 4).

Since the 2014 guideline update advised against using palivizumab for preterm infants born at or after 29 wGA without additional risk factors, nirsevimab has emerged as a new option for this demographic. In 2024, Yu T and colleagues adopted a hybrid decision tree-Markov model to evaluate the cost-effectiveness of RSV prophylaxis with nirsevimab and palivizumab in infants born between 29 and 34 weeks and 6 days’ gestational age in the United States. Their findings revealed that, compared to no prophylaxis, palivizumab incurred an additional cost of USD 9572 from a healthcare perspective and USD 9584 from a societal perspective, leading to incremental cost-effectiveness ratios exceeding USD 5 million per QALY from both viewpoints. According to Phase IIB trial data, threshold analysis suggested that nirsevimab would be deemed cost-effective if its price were set underused $1923 from a healthcare standpoint and under USD 1962 from a societal perspective. Additionally, in direct comparison with palivizumab, nirsevimab would maintain cost-effectiveness at any pricing below USD 11,211 from a healthcare angle and below USD 11,263 from a societal viewpoint [83]. In a modeled head-to-head comparison of nirsevimab and the maternal RSV vaccine for protecting infants during their first RSV season, data from the Medley, Phase IIB, MELODY, and HARMONIE trials indicated that nirsevimab could prevent a total of 291,320 events compared to the SoP. With the same coverage rate of 80%, the maternal vaccine would prevent approximately half as many events, totaling 144,278, based on data from the Matisse trial [84]. In a population-based modeling study conducted in Spain, real-world data from PC visits and hospital admissions were examined. The study assessed a seasonal catch-up immunization strategy using nirsevimab, which assumed an initial effectiveness of 79.5% for the first 5 months, gradually decreasing linearly to 0% by the 10th month. This approach could prevent 5121 to 8846 cases of RSV bronchiolitis per 100,000 infant-years. With an effectiveness rate of 77.3% and the same decay pattern, the strategy could avert 976 to 1686 RSV-related hospitalizations per 100,000 infant-years, depending on the vaccination coverage. Furthermore, a year-round maternal RSV vaccine with an initial effectiveness of 51% during the first 6 months, declining linearly to 0% by month 10, could prevent 3246 to 5606 cases of RSV bronchiolitis per 100,000 infant-years. Assuming an effectiveness of 56.9% and following the same decay pattern, this vaccine could reduce RSV-related hospitalizations by 713 to 1231 per 100,000 infant-years [85]. The economic benefits of combining nirsevimab administration with maternal RSVpreF vaccination to protect infants against RSV disease have been evaluated. Research in Canada employed a discrete-event simulation model to examine data regarding RSV infections that required medical attention in infants under 1 year of age during the period from 2010 to 2019. The investigation compared two immunization strategies: (1) administering nirsevimab to all newborns regardless of risk factors, and (2) a combined approach involving year-round maternal vaccination alongside nirsevimab for infants with high risk during the RSV season. When applying a willingness-to-pay threshold of CAD 50,000 QALY gained, the analysis indicated that the nirsevimab-only strategy would be cost-effective from a societal standpoint if priced at up to CAD 290 per dose, resulting in an annual budget impact of CAD 83,978 for every 1113 infants out of 100,000 people. Conversely, the integrated strategy would incur a lower budget impact of CAD 49,473 per 100,000 population, assuming a dose price of CAD 290 for nirsevimab and CAD 195 for the RSVpreF vaccine. This comprehensive approach would reduce infant mortality by 76–85%, a rate similar to the 78% reduction observed when nirsevimab alone is given to the entire population of newborns [86].

In future studies, it is essential to employ dynamic models to further evaluate the cost-effectiveness of nirsevimab. Additionally, a deeper analysis should be conducted to understand how nirsevimab influences respiratory virus trends, particularly in terms of hospitalization rates and severe infection rates. Long-term outcomes of nirsevimab use should also be monitored and analyzed. It is crucial for guiding decisions about nirsevimab medication management.

## 7. Recommendations for the Application of Nirsevimab

Based on clinical trials and real-world evidence, the administration of nirsevimab for infants under 2 years old is guided by their age, weight, and risk factors. The ACIP recommends a single dose of nirsevimab for all infants younger than 8 months: those weighing less than 5 kg should receive 50 mg, while infants weighing 5 kg or more should be given 100 mg. For high-risk infants aged between 8 and 19 months entering their second RSV season, a single dose of 200 mg is advised, administered as two concurrent 100 mg injections at separate sites. High-risk factors include chronic lung disease in preterm infants who have required medical interventions within 6 months before the second RSV season; severe immunodeficiency; cystic fibrosis with significant lung involvement or a weight-for-length below the 10th percentile; and children who are American Indian or Alaska Native [87,88]. The Spanish Neonatology Association has issued recommendations for the 2024–2025 RSV season. For healthy preterm infants with GA exceeding 35 weeks and less than 6 months old, a single dose of nirsevimab should be administered at the onset of the RSV season. For preterm infants born prior to 35 wGA and under 12 months old, nirsevimab is suggested as an alternative to palivizumab. It was recommended that infants with high-risk factors receive one dose of prophylaxis at the start of each RSV season until they reach 24 months old. These dosing guidelines were in accordance with the recommendations from the ACIP [89]. Nirsevimab is currently available in two dose strengths: 50 mg/0.5 mL and 100 mg/1 mL, both provided in pre-filled syringes for single-use administration. Pre-filled syringes should be stored under refrigerated conditions between 36 °F to 46 °F (2 °C to 8 °C), protected from light. They can also be stored at room temperature (68 °F to 77 °F or 20 °C to 25 °C) for up to 8 h. It is important to avoid freezing or exposure to excessively hot environments. Once removed from refrigeration, the medication must be used within 8 h; otherwise, it should be discarded. Expired nirsevimab should not be administered. Nirsevimab is intended for intramuscular injection, with the preferred injection site being the anterolateral aspect of the thigh [90].

Nirsevimab can be administered concurrently with other vaccines, and there is no required specific time interval between its administration and that of live vaccines. It is anticipated that nirsevimab will not interfere with the immune response elicited by vaccine products. However, experience with the concurrent use of nirsevimab and vaccines is currently limited. Notably, in clinical trials, when nirsevimab was co-administered with routine childhood vaccines, it did not increase safety concerns or reactogenicity [91].

## 8. Implementation Challenges of Nirsevimab Immunization

Novel RSV immunizations with nirsevimab show significant potential to reduce the RSV burden in infants, thereby substantially impacting clinical practice. To facilitate their effective use and ensure all children receive adequate protection at the onset of their first RSV season, an ongoing partnership among parents, healthcare professionals—especially those in PC, obstetrics, pediatrics—and public health authorities must be strengthened. The mAb should be accessible, affordable, and approved by national regulatory authorities. Notably, nirsevimab is the only mAb included in the VFC program. Effective communication between healthcare providers and parents, as guardians of infants, is essential for addressing any hesitations regarding the use of mAb. Various factors such as RSV seasonality, cost-effectiveness, cultural context, and health policies influence the preferred nirsevimab administration strategies in different countries. For the 2023–2024 RSV season, France and Spain implemented seasonal mAb programs [92,93], while the United Kingdom adopted a year-round approach using either mAb or maternal RSV vaccination [94]. In the United States, the Centers for Disease Control (CDC) recommends seasonal use of nirsevimab or maternal RSV vaccination without preference [90].

Compared to palivizumab, nirsevimab is significantly less expensive but remains the most costly childhood vaccination recommended by the CDC. The cost per dose in the private sector is USD 495, while it is USD 395 through the VFC program [95]. Despite its high cost, nirsevimab experienced supply shortages during the 2023–2024 RSV season. On 17 October 2023, orders for nirsevimab through the VFC program were suspended due to drug shortages [96]. Subsequently, on 23 October 2023, the CDC announced limited availability of the 100 mg formulation and updated its recommendations for nirsevimab use. Infants with high-risk factors were prioritized for the 100 mg dose, and administration of nirsevimab to children aged 8–19 months who qualify for palivizumab treatment was temporarily halted [97]. Additionally, there are other considerations regarding the use of two 50 mg doses of nirsevimab as an alternative to a single 100 mg dose; however, this approach has not been approved or recommended [97]. The American Academy of Pediatrics (AAP) suggested incorporating nirsevimab into bundled payments for newborn hospitalizations to alleviate the financial strain on medical centers and ensure timely administration to infants born during the RSV season [95]. Furthermore, it is crucial to provide early support to pediatric clinics and healthcare institutions for the procurement of nirsevimab and to assist them during the reimbursement waiting period. To aid in making purchasing decisions, the AAP recommends that hospitals and clinics evaluate their payer mix, cash flow status, and anticipated acceptance of nirsevimab by families [98].

A research project was conducted using electronic health record data from a statewide primary care network in Massachusetts. The analysis covered data from 79 practices serving around 400,000 children during the timeframe from 7 October 2023 to 30 November 2023. It was observed that practices offering nirsevimab tended to be larger than smaller ones, with RRs of 2.12 (95% CI: 1.15–3.92) for medium-sized practices and 2.12 (95% CI: 1.16–3.90) for large practices. Practices providing nirsevimab had a smaller percentage of patients with public insurance compared to those with commercial insurance (RR = 0.97). Furthermore, these practices also had fewer patients residing in lower-income ZIP codes. Specifically, when compared to the highest income quartile, the RRs for the second to fourth quartiles were 0.93, 0.89, and 0.86, respectively. Among infants eligible for nirsevimab, 47.3% received it. Factors that were negatively associated with receiving nirsevimab included older age (RR = 0.98 per week increase), public insurance (RR = 0.76), and living in lower-income ZIP codes. Specifically, compared to the highest income quartile, the third and fourth quartiles had RRs of 0.90 [0.81–0.99] and 0.82 [0.67–0.99], respectively. Factors positively associated with receiving nirsevimab immunization included having a preferred language other than English (RR = 1.10) and the presence of chronic diseases. Specifically, the RR was 1.14 for individuals with noncomplex chronic diseases and 1.24 for those with complex chronic diseases, compared to individuals without chronic diseases [99]. The VFC program also provides free nirsevimab immunization to the following groups: uninsured children, underinsured children in certain circumstances, children enrolled in Medicaid, or American Indian and Alaska Native children [100]. At the onset of the nirsevimab promotion, 86% of pediatrician offices were enrolled in the VFC program, whereas only 10% of birthing hospitals participated [101]. A retrospective analysis of medical records was performed for all newborns discharged from the newborn nursery and NICU at an urban academic medical center in the Bronx during the period from December 2023 to March 2024. Out of the 312 newborns examined, 92.3% (288) qualified for the VFC program. Among these eligible infants, 55.7% (160) received nirsevimab, while 11.1% (32) had birthing parents who received the RSVpreF vaccine [102]. In a separate review of hospital-discharged newborns between December 2023 and February 2024, it was observed that 57% of the 1326 infants received nirsevimab. Key factors significantly linked to receiving nirsevimab included having public or alternative insurance (rather than private insurance), speaking a primary language other than English, being a first-time parent, and receiving ocular prophylaxis along with the hepatitis B vaccine [103].

The understanding and perspectives of parents regarding immunization play a crucial role, as they are responsible for ensuring their children receive essential vaccinations. A cross-sectional observational study was conducted with 3217 Spanish parents or guardians of children under 2 years old, concluding on 1 September 2023. The results indicated that nearly all participants (95.8%) were familiar with bronchiolitis, whereas awareness of RSV was significantly lower at 46.6%, mostly acquired after the birth of their first child. Information about RSV and bronchiolitis was primarily sourced from family members, with only a small fraction (4.8%) receiving this information from healthcare providers. Knowledge of nirsevimab was limited to just 11.2% of respondents. Concerns surrounding nirsevimab mainly revolved around safety and possible side effects, while those who were not informed expressed dissatisfaction with the lack of information on RSV infection [104]. These findings highlight the critical need for more effective educational initiatives aimed at all parents and legal guardians.

Ensuring that eligible infants receive nirsevimab requires a multidisciplinary approach. This procedure is shaped by multiple factors such as national vaccination guidelines, medical care routines, choices made by caregivers, and the accessibility of nirsevimab. Healthcare providers specializing in pediatrics can provide crucial perspectives and firsthand feedback from patients and their families to inform policy decisions. Government bodies should focus on assisting pediatricians, especially when there are drug shortages. Moreover, pediatric specialists can work together with department heads, healthcare workers, pharmacists, and IT professionals to optimize workflow processes. As the distribution of nirsevimab continues to expand, it is vital to conduct additional research to assess its economic viability and overall benefits.

## 9. Prospects and Potential of Nirsevimab

RSV infections remain a substantial global health concern because of their widespread occurrence and the complications they cause, especially in infants. The pervasive nature of this virus and its significant impact on healthcare systems underscore the urgent need for comprehensive vaccination approaches. Although progress in mAb therapies provides notable protection, the high expenses associated with these treatments restrict their broad application, particularly in areas with limited resources.

Nirsevimab offers significant potential to reduce the impact of severe RSV infections among young children. However, its broad implementation depends on various factors, including national immunization guidelines, pediatric specialists’ recommendations, caregivers’ choices, and economic considerations. Continuous monitoring and improvements in deployment strategies are crucial to maximizing its effectiveness and maintaining sustainability across different healthcare environments. In developing nations such as China, nirsevimab’s introduction is currently restricted to large urban centers, with gradual and limited expansion. It has not yet been incorporated into the national vaccination schedule. Public awareness regarding RSV risks and the importance of early intervention remains low, and understanding and acceptance of nirsevimab among caregivers are still insufficient. Although substantial high-quality research exists, most studies have concentrated on infants under 2 years, particularly those aged 0–6 months. To provide optimal protection during the first RSV season, it is advisable to administer nirsevimab to newborns shortly after birth or prior to hospital discharge, ensuring coverage for up to 5 months. A follow-up dose should be given during the subsequent RSV season. Data on newborn immunizations are scarce, with only limited information available from countries like Spain, where such data exist but lack detailed analysis.

## 10. Conclusions

This article comprehensively overviews the clinical application of nirsevimab in infant populations based on current evidence. As the predominant pathogen causing bronchiolitis and lower respiratory tract infections in infants, RSV poses significant clinical burdens. The monoclonal antibody nirsevimab demonstrates high efficacy and safety—adverse events match a placebo, with no serious treatment-related AEs. A single dose provides protection throughout the entire RSV season, cutting RSV hospitalization risks by ~80% and reducing severity. While cost-effectiveness data support universal immunization in infants to alleviate healthcare and economic burdens, implementation hurdles persist, including policy frameworks, supply chain logistics, pricing structures, and caregiver hesitancy.

## Figures and Tables

**Table 1:** Adverse events of nirsevimab in clinical trials.

AEs\Trials		Phase 2B Trial 2020 [37]		MELODY (Phase 3 Trial) 2022 [39]		HARMONIE (Phase 3B, Pragmatic, Open-Label Trial), 2023 [42]	
	Group	Nirsevimab (*n* = 968)	Placebo (*n* = 479)	Nirsevimab (*n* = 987)	Placebo (N = 491)	Nirsevimab (*n* = 4015)	SoP (*n* = 4020)
AEs	any	834 (86.2)	416 (86.8)	863 (87.4)	426 (86.8)	1479 (36.8)	1326 (33.0)
	related to trial drug	22 (2.3)	10 (2.1)	10 (1.0)	7 (1.4)	86 (2.1)	0
	≥ Grade 3	77 (8.0)	60 (12.5)	36 (3.6)	21 (4.3)	48 (1.2)	0
	within 30 min	-	-	-	-	26 (0.6)	0
	within 24 h	24 (2.5)	12 (2.5)	18 (1.8)	3 (0.6)	-	-
	within 3 days	-	-	56 (5.7)	23 (4.7)	-	-
	within 7 days	121 (12.5)	73 (15.2)	132 (13.4)	63 (12.8)	-	-
Death		2 (0.2)	3 (0.6)	3 (0.3)	0	0	0
SAEs	any	108 (11.2)	81 (16.9)	67 (6.8)	36 (7.3)	89 (2.2)	67 (1.7)
	related to trial drug	0	0	0	0	1 (<0.1)	0
AEs of special interest	any	5 (0.5)	3 (0.6)	1 (0.1)	0	3 (0.1)	1 (<0.1)
	related to trial drug	5 (0.5)	3 (0.6)	-	-	-	-

AEs: adverse events, SAEs: severe adverse events, SoP: standard of practice.

**Table 2:** Effectiveness of nirsevimab for RSV infection in clinical trials.

Clinical Trials	Time	Population	Main Results
Phase 2B trial 2020 [38]	November 2016–November 2017	Infants born preterm (29–34^+6^ wGA) Nirsevimab, *n* = 969 Placebo, *n* = 484	Compared to the control, the nirsevimab group experienced a 70.1% (95% CI: 52.3–81.2%) reduction in the incidence of RSV-MALRTI (9.5% [46 infants] vs. 2.6% [25 infants]), and a 78.4% (95% CI: 51.9–90.3%) decrease in RSV-LRTI-H (4.1% [20 infants] vs. 0.8% [8 infants]).
MELODY (Phase 3 trial) 2022 [39]	At 150 northern-hemisphere (2019) and 10 southern-hemisphere (2020) sites.	Infants born ≥35 wGA Nirsevimab, *n* = 994 Placebo, *n* = 496	Compared to the placebo, the nirsevimab group experienced a 74.5% (95% CI: 49.6–87.1) reduction in RSV-MALRTI (5% [25 infants] vs. 1.2% [12 infants]) and a 62.1% (95% CI: −8.6 to 86.8) decrease in RSV-LRTI-H (1.6% [8 infants] vs. 0.6% [6 infants]).
HARMONIE (Phase 3B, pragmatic, open-label trial) 2023 [42]	8 August 2022–28 February 2023	Infants born ≥29 wGA Nirsevimab, *n* = 4037 SoP, *n* = 4021	Compared to the SoP, nirsevimab was 83.2% effective (95% CI: 67.8–92.0) in preventing RSV-LRTI-H (1.5% [60 infants] vs. 0.3% [11 infants]), and 75.7% effective (95% CI: 32.8–92.9) in preventing very severe RSV-LRTI (0.5% [19 infants] vs. 0.1% [5 infants]).

SoP: standard of practice, RSV-MALRTI: medically attended RSV-LRTI, RSV-LRTI-H: RSV-LRTI related hospitalization.

**Table 3:** Effectiveness of nirsevimab for RSV infection in real world.

Study Design	Study Details	Population	Main Results
Systematic Review and Meta-Analysis	Riccò M, et al., 2024 [47]. Nirsevimab for RSV-H prevention	19 series (5 RCTs, 7 real-world reports, 1 health authority report), *n* = 45,238	Pooled effectiveness in preventing RSV-H was 88.4% (95% CI: 84.7–91.2%).
Population-based, 3-year longitudinal study, interim results (Galicia, Spain)	Ares-Gómez S, et al., 2024 [29]. RSV-LRTI-H, RSV-LRTI ICU admissions	Infants born between 25 September 2023 and 31 March 2024, *n* = 10,259. 14 hospitals	nIC with Nirsevimab: 81.4–97.5%. Nirsevimab showed 82.0% effectiveness against RSV-LRTI-H (immunized: 0.3% [30/9408] vs. non-immunized: 1.9% [16/851]), with 86.9% effectiveness (95% CI: 69.1–94.2) for preventing ICU admissions (immunized: 0.16% [15/9408] vs. non-immunized: 1.18% [10/851]), preventing 407 RSV-LRTI-H cases per 1000 infants (89.8% effectiveness).
Population-based study (France)	Jabagi MJ, et al., 2025 [53] RSV-H, Severe hospitalization outcomes	All infants born between 6 February and 15 September 2023, *n* = 82,474 (1:1 ratio)	Nirsevimab reduced RSV-LRTI-H by 65% (0.8% [342] vs. 2.4% [992]). Effectiveness in reducing severe outcomes was: 74% for PICU, 64% for HDU, 66% for ventilation, and 67% for oxygen therapy.
Population-based cohort study (Navarre, Spain)	Ezpeleta G, et al., 2024 [30]. RSV-ER attention, RSV-H, RSV-ICU admission	All infants born from October to December 2023, *n* = 1177. 1 region	nIC with Nirsevimab was 92.0%. RSV-H risk: 0.7% (8/1083) immunized vs. 8.5% (8/94) non-immunized. Effectiveness in preventing RSV-ER attendance: 87.9% (95% CI: 70.3–95.1); RSV-H: 88.7% (95% CI: 69.6–95.8); RSV-ICU admission: 85.9% (95% CI: 13.2–97.7). Immunization at birth prevented one hospitalization per 15.3 infants treated.
Multicenter hospital-based active surveillance, interim results (Spain)	López-Lacort M, et al., 2024 [28]. RSV LRTI-H in infants < 9 months.	Infants in their first RSV season (born on or after 1 April 2023), *n* = 15,676. 9 hospitals, 3 regions	nIC with Nirsevimab: 78.7–98.6%. RSV-LRTI-H prevention: 70.2% efficacy (38.3–88.5), varying by Site: Valencia: 69.3%, Murcia: 86.9%, Valladolid: 97.0%. LRTI-negative RSV admissions: 32.4% efficacy (−27.5–63.4).
Retrospective cohort study (Catalonia, Spain)	Coma E, et al., 2024 [31]. RSV infection, PC attended bronchiolitis, Viral Pneumonia, ER Visit, Hospital and ICU admission.	Infants born between April and September 2023, *n* = 26,525. All hospitals, 1 region (Catalonia)	nIC with Nirsevimab: 23,127/26,525 (87.2%). Risk reduction: RSV Infection: 68.9%, PC attended bronchiolitis: 48.1%, Viral Pneumonia: 60.7%, ER Visit: 55.4%, RSV-H admission: 87.6%, RSV-ICU admission: 90.1%.
Observational, multicenter, prospective study (Spain)	Rius-Peris JM, et al., 2025 [54] RSV-H	Infants <12 months, 1 September 2021–15 June 2024, *n* = 2656, 20 hospitals	The 2023–2024 season saw a 20–30% reduction in RSV-bronchiolitis hospitalizations compared to 2021–2023, with nirsevimab demonstrating 70% effectiveness in preventing RSV-H.
Prospective, descriptive, observational study (Spain)	Alejandre C, et al., 2024 [55] RSV-PICU admissions, September 2010–February 2024	PICU-admitted severe bronchiolitis patients, *n* = 1531. Median age: 52 days (IQR 28–104).	The total number of PICU bronchiolitis admissions was significantly lower in the post-nirsevimab period (73 vs. 1458, *p* = 0.03).
Test-negative study (France)	Lenglart L, et al., 2025 [56] Effectiveness in the PED	All infants <1 year with first-time bronchiolitis in the PED, *n* = 383	Nirsevimab immunization: 9.8% (27/274) in cases vs. 46.2% (50/109) in controls; effectiveness 82.5% (68.0–90.8) at PEDs.
Test-negative case-control study (Spain)	Agüera M, et al., 2024 [57] RSV-LRTI	Infants <12 months, *n* = 234. 3 centers	RSV was detected in 60.2% (141/234) of cases, with lower prevalence in immunized (37%) versus non-immunized (75%) groups. Effectiveness against RSV-LRTI was 81.0% (95% CI: 60.9–90.7) and 85.6% (41.7–96.4) for severe disease (NIV/CMV requirement).
Case control study (France) (NCT06185647)	Carbajal R, et al., 2024 [58] RSV-related ED visits, all-cause bronchiolitis hospitalizations, RSV-H	All infants ≤24 months attended in PED, *n* = 2786	All-cause bronchiolitis cases (*n* = 864) vs. controls (*n* = 1922). Nirsevimab prevented: 83% of RSV-related bronchiolitis ED visits; 59% of all-cause bronchiolitis hospitalizations; 83% of RSV-associated bronchiolitis hospitalizations; 91% of oxygen-requiring cases; 88% of cases needing nasogastric feeding.
Single-center, observational study (Spain)	Molina Gutiérrez M, et al., 2024 [59] PED attendance. Between weeks 40 and 52 of 2022 and 2023	Infants with RSV-confirmed infection in the PED, *n* = 765	PED attendance was 80.3% in 2022 and 19.7% in 2023 (nirsevimab immunization campaign began in October 2023).
Retrospective study (Spain)	Jimeno Ruiz S, et al., 2024 [60]. RSV-H, Pre-/post-COVID: 2018–2019, 2019–2020, 2022–2023, 2023–2024	Children under 6 months of age, between 1 October and 31 March across four seasons.	During the 2023/2024 season following nirsevimab introduction, RSV-LRTI hospitalizations dropped by 79.3% in infants <3 months and by 66.9% in those aged 3–6 months.
Retrospective study (France)	Marouk A, et al., 2025 [61] RSV-H, RSV-PICU admissions, RSV positivity	Infants aged <3 months with bronchiolitis, *n* = 737	Among 737 cases, 531 (72%) received nirsevimab, and 402 (54%) were hospitalized. Nirsevimab showed efficacy rates of 53.5% for preventing hospitalization, 51.1% for preventing PICU admissions, and 79.6% for preventing RSV positivity.
National multicenter prospective study (OVNI), France	Jeziorski E, et al., 2025 [62] RSV-positive bronchiolitis	All infants ≤12 months hospitalized due to acute bronchiolitis, *n* = 1105	14.3% (102/711) of RSV-positive cases received nirsevimab, while 44.9% (128/285) of RSV-negative cases received nirsevimab. Nirsevimab demonstrated 79.5% effectiveness against RSV-positive bronchiolitis [14.3% (102/711 cases) vs. 44.9% (128/285 controls)].
Multicenter case-control observational study (Western Australia)	Wadia U, et al., 2025 [63]. RSV-H	Hospitalized children with lab-confirmed RSV-ARI versus test-negative controls, *n* = 284	Nirsevimab coverage was 22.8% in RSV cases versus 60.0% in controls, with 88.2% adjusted effectiveness against RSV-ARI hospitalization.
Retrospective observational cohort study	Moreno-Pérez D, et al., 2025 [64] During the 2023–2024 nirsevimab immunization campaign	All hospitalizations of infants <6 months due to RSV, *n* = 222	Nirsevimab exposure reduced: Nasal cannula use by 64%, MV/NIV use by 48%, PICU admission by 54%, Hospitalization duration by 30%, Nasal cannula duration by 31%.
Age-structured deterministic model study (France)	Brault A, et al., 2024 [65]. RSV-H, 2023–24 RSV season	Infants <24 months	Nirsevimab prevented 5800 RSV-bronchiolitis hospitalizations (23% reduction, 95% CI 16–30), with greater efficacy in infants aged 0–2 months (4200 prevented; 35% reduction, 25–44). At 215,000 administered doses, effectiveness was 73%, requiring 39 doses to prevent one hospitalization.
Pre-post ecological study (Spain)	Gregori-García E, et al., 2025 [66] RSV-H	All cases of RSV infection in children <5 years, *n* = 489. 2022–23 season vs. 2023–24 season (nirsevimab recommended)	Nirsevimab in 2023–24 cut RSV risk in <6mo infants (RR = 0.16) but not 6–11mo (RR = 0.90). Hospitalized <6mo: greatest protection (RR = 0.13).
Test-negative case-control study (US)	Xu H, et al., 2024 [67]. RSV infection, outpatient visits, RSV-H, RSV-ICU	*n* = 3090 (median age 6.7 months, IQR 3.6–9.7) 1 October 2023–9 May 2024	3.1% (21/680) RSV-positive and 12.8% (309/2410) RSV-negative infants received nirsevimab. Efficiency in preventing: RSV infection 68.4% (95% CI: 50.3–80.8%), outpatient visits 61.6% (95% CI: 35.6–78.6%), RSV-H 80.5% (95% CI: 52.0–93.5%), RSV-ICU admission 84.6% (95% CI: 58.7–95.6%).
Test-negative case-control study (France)	Lassoued Y, et al., 2024 [68]. RSV-bronchiolitis in outpatients	Infants <12 months, *n* = 883, 15 September 2023–1 February 2024	13.7% (62/453) of case patients and 41.2% (177/430) of control patients received nirsevimab. Effectiveness against RSV bronchiolitis: 79.7% (95% CI 67.7–87.3).
Test-negative study (Spain)	López-Lacort M, et al., 2025 [69]. MA-LRTI	Infants <10 months from 57 PC centers, *n* = 160	Nirsevimab coverage: 88% (141/160) infants, RSV-positive infants: 44 (27.5%); effectiveness against MA- LRTI: 75.8% (95% CI: 40.4–92.7). Catch-up group: 128 infants received nirsevimab; RSV-positive infants: 37 (28.9%); effectiveness against MA- LRTI: 80.2% (95% CI: 44.3–95.4).
Test-negative case-control study (France)	Paireau J, et al., 2024 [34]. RSV-PICU admissions	Infants <1 month, or <5 months with comorbidities. 15 September 2023–31 January 2024. *n* = 288	263 (91%) infants were 0–3 months old. Nirsevimab showed 75.9% (95% CI: 48.5–88.7) effectiveness against RSV-bronchiolitis PICU admissions. In the sensitivity analyses, the nirsevimab effectiveness was 80.6% (95% CI: 61.6–90.3) and 80.4% (95% CI: 61.7–89.9).
Data from new vaccine surveillance network	Moline HL, et al., 2024 [32]. RSV-H	Infants born after 1 October 2023, or <8 months by 1 October 2023, *n* = 699. October 2023–February 2024	nIC with Nirsevimab: high-risk conditions 46% (18/39); no risk conditions 6% (41/660). Reduction in RSV-H: 90%. Time elapsed between nirsevimab administration and ARI onset: 7–127 days; median = 45 days (IQR = 19–76 days).
Single-center study (Luxembourg)	Ernst et al., 2024 [33]. RSV-H in children ≤5 years	Infants born between 1 October 2023 and 31 March 2024, *n* = 1524. 4 hospitals, the entire country	nIC with Nirsevimab from Oct to mid-Dec: 1277/1524 (84%). RSV-H reduction: children ≤5 years, −38%; infants <6 months, −69%. PICU hospitalization proportion (<5 years): 2022, 36/389 (9.3%); 2023, 15/241 (6.2%). Average age of hospitalized children: 7.8 months (2022) vs. 14.4 months (2023).

MA-LRTI: medically attended LRTI; RSV-H: RSV-related hospitalization; RSV-LRTI-H: RSV-LRTI pediatric hospitalization; nIC: neonatal immunization coverage; ARI: acute respiratory illness; PED: pediatric emergency department; HDU: high dependency unit; NIV: non-invasive ventilation, MV: invasive mechanical ventilation.

**Table 4:** Cost-effectiveness of nirsevimab for RSV prophylaxis.

Settings	Year	Population	Nirsevimab’s Impact on RSV-Related Health Events and Costs	
			Under the SoP	Universal immunization
Static decision-analytic model	Kieffer A, et al., 2022 [73]	US birth cohort during its first RSV season	RSV resulted 529,915 RSV-MALRTIs, 47,281 RSV-H, representing USD 1.2 billion (2021 US dollars [USD]) in costs.	Reduce 290,174 RSV-MALRTI, 24,986 RSV-H, and saving USD 612 million 2021 USD.
Static decision-analytic model	Gil-Prieto R, et al., 2024 [74]	Spanish birth cohort during its first RSV season	RSV resulted in 151,741 PC visits, 38,798 ER visits, 12,889 RSV-H, 1412 PICU admissions, and 16 deaths, with an associated cost of EUR 71.8 million.	Prevent 97,157 PC visits (64.0% reduction), 24,789 ER visits (63.9%), 8185 RSV-H (63.5%), 869 PICU admissions (61.5%), and 9 inpatient deaths (52.6%), saving EUR 47.8 million (62.4%).
Static modeling study	Alharbi A, et al., 2024 [75]	All infants in their first RSV season in the KSA	RSV resulted in 17,179–19,607 RSV-H, 2932–3625 PICU and 172–525 MV cases, 57,654–191,115 ER visits, 219,053–219,970 PC visits, 14 deaths, 12,884–14,705 cases of recurrent wheezing, with an associated cost of SAR 480–619 million.	Reduce 58% of RSV-H (58% PICU cases, 58% MV episodes), 53% of ER visits, 53% of PC visits, 58% of episodes of recurrent wheezing, 8 deaths, saving SAR 274–343 million.
Static decision-analytic model	Kieffer A, et al., 2024 [77]	Infants aged <1 year in their first RSV season (UK)	RSV led to 329,425 LRTIs, including 24,381 severe cases (hospitalizations/ICU), with costs of GBP 117.8M (2024 GBP).	Averting 198,886 RSV LRTIs (16,657 severe cases) could reduce treatment costs by GBP 77.2 million.
Static decision-analytic model	Marcellusi A, et al., 2025 [78]	All infants in their first RSV season (Italy)	RSV led to 216,100 RSV-LRTIs, 15,121 complications, and 16 deaths, costing EUR 64.4M (EUR 50.5 M treatment, EUR 10.9M complications, EUR 3 M productivity loss)	Preventing 100,208 RSV-LRTIs, 6969 complications, and 6 deaths could save EUR 23.3M (treatment), EUR 5M (complications), and EUR 1.2M (productivity loss).
Static decision-analytic model	Noto S, et al., 2025 [79]	All infants in Japan	RSV-associated healthcare costs: ICU admission: 2,022,198; mechanical ventilation: JPY 270,899; ER visit: JPY 10,613; hospitalization: JPY 391,648; primary care visit: JPY 2910.	At JPY 45,000 per dose, nirsevimab reduces approximately 50% of RSV-related health events in infants. From a societal perspective, the ICER decreases to JPY 1,695,635/QALY.
A static cost-effectiveness model	Zeevat F, et al., 2025 [80]	All infants during their first RSV season in the Netherlands	RSV resulted in 14,844 NMA cases, 19,373 GP visits, 3197 hospitalizations, and 204 PICU admissions.	Preventing 9574 NMA cases, 12,663 GP visits, 2333 hospitalizations, and 150 PICU admissions.
Static decision tree model	Shin T, et al., 2025 [81]	Canadian infant birth cohort	RSV caused 138,981 PC visits, 40,254 ER visits, 5532 hospitalizations (including 1328 ICU admissions), 320 ventilated cases, and 16 in-hospital deaths.	At ~80% coverage, nirsevimab immunization could prevent 34,672 GP visits, 10,633 ER visits, 2296 hospitalizations, and 7 deaths, saving CAD 70 million.
Decision analytic model	Hutton DW, et al., 2024 [82]	Secondary data simulated the short- and long-term effects of RSV in infants. At 50% coverage of the US birth cohort.		Preventing 107,253 outpatient visits, 38,204 ED visits, and 14,341 hospitalizations annually at a cost of USD 308,468 per QALY.

RSV-H: RSV-related hospitalization, RSV-MALRTI: medically attended RSV-LRTI, SoP: standard of practice.

## Abbreviations

The following abbreviations are used in this manuscript:

AAP	American Academy of Pediatrics
ACIP	Advisory Committee on Immunization Practices
AEs	adverse events
CDC	Centers for Disease Control
CI	confidence interval
ER	emergency room
FI-RSV	formalin-inactivated RSV vaccine
GA	gestational age
GP	general practitioner
HDU	high dependency unit
ICU	intensive care unit
LRTIs	lower respiratory tract infections
mAb	monoclonal antibody
MV	invasive mechanical ventilation
NIV	non-invasive ventilation
PC	primary care
PED	pediatric emergency department
PICU	pediatric intensive care unit
QALY	quality-adjusted life year
RR	relative risk
RSV	respiratory syncytial virus
SAEs	severe adverse events
SoP	standard of practice
VFC	Vaccines for Children
wGA	weeks gestational age
